# Effects of phytocannabinoids, synthetic and semi-synthetic cannabinoids on microglia and astrocytes: Review of neuroinflammatory mechanisms

**DOI:** 10.1093/jnen/nlag049

**Published:** 2026-05-15

**Authors:** Giulia Brambilla, Giacomo Belli, Andrea Paggi, Luca Morini, Silvia Damiana Visonà

**Affiliations:** Department of Public Health, Experimental and Forensic Medicine, University of Pavia, Pavia, Italy; Department of Public Health, Experimental and Forensic Medicine, University of Pavia, Pavia, Italy; Oxford Health NHS Foundation Trust, Oxford, United Kingdom; Department of Public Health, Experimental and Forensic Medicine, University of Pavia, Pavia, Italy; Department of Public Health, Experimental and Forensic Medicine, University of Pavia, Pavia, Italy

**Keywords:** glial cells, neuroinflammation, neuroprotection, phytocannabinoids, semi-synthetic cannabinoids, synthetic cannabinoids

## Abstract

Over 120 phytocannabinoids and 190 synthetic and semi-synthetic cannabinoids have been identified. Many of these currently circulate in recreational and illicit drug markets. Epidemiological evidence indicates a progressive increase in their use but long-term effects of cannabinoids on the CNS remain poorly understood. Exogenous cannabinoids can interact with cannabinoid receptor CB2, which is expressed on astrocytes and microglia, the key regulators of neuroinflammatory responses. Dysregulated or chronic microglial activation can sustain neuroinflammation, a central mechanism underlying neurodegenerative diseases. Clarifying cannabinoid-induced alterations in glia is therefore crucial both because their widespread consumption and the global burden of neurodegenerative disorders, for which cannabinoids might offer therapeutic potential. This review was conducted by a multidisciplinary team following JBI and PRISMA-ScR guidelines that systematically mapped available evidence across PubMed, Scopus and Web of Science. The findings are thematically organized and qualitatively summarized and indicate compound and time-dependent effects. Acute exposure appears to be neuroprotective whereas chronic effects remain unclear. Preliminary data suggest that some synthetic and semi-synthetic cannabinoids may retain protective actions while Δ9-tetrahydrocannabinol (THC) may promote glial activation and neuroinflammation, These results underscore the need for further in vivo and longitudinal studies to evaluate long-term impacts and inform safe therapeutic and regulatory strategies.

## Introduction

Preparations derived from *Cannabis sativa* have been consumed for centuries for both medicinal and more commonly recreational purpose (1). Among its bioactive compounds, more than 120 phytocannabinoids have been identified, with cannabidiol (CBD) and Δ9-tetrahydrocannabinol (THC) being the most extensively studied. THC is responsible for the psychoactive effects of cannabis which, according to the last World Drug Report by the United Nations Office on Drug and Crime (UNODC), is the most widely used psychoactive substance worldwide, with approximately 219 million users in 2021, and the highest prevalence observed among adolescents (2).

Epidemiological data indicate a rising trend not only in overall use but also in daily consumption among both adults and adolescents, particularly in regions where legalization has increased accessibility. In the United States, where many states have legalized cannabis for recreational and/or medical purposes, 15.3% of adults (18–64 years) reported current cannabis use, with 7.9% classified as daily or near-daily users, according to the 2022 Behavioral Risk Factor Surveillance System (BRFSS) (3). Similarly, the Monitoring the Future (MTF) survey estimated that daily or near-daily cannabis use among high-school students (14–18 years) was 4.1% in 2020, compared with just under 1% in 1991 (4). Although frequent cannabis use is less prevalent in Europe where most countries maintain restrictive policies, use remains significant: the 2023 European Drug Report indicates that about 8.4% of individuals aged 15–64 used cannabis in the past years, with approximately 1.5% reporting daily or near-daily users (5). Moreover, cannabis potency on the illicit market has increased markedly over time, as shown by the Drug Enforcement Administration (DEA), which reported an average THC content of 3.96% in 1995, rising to 16.14% in 2022 (6).

In addition to natural cannabinoid, (also known as phytocannabinoids), synthetic and semi-synthetic cannabinoids have emerged as a major concern. Semi-synthetic cannabinoids are chemically modified derivatives of natural compounds whereas synthetic cannabinoids are entirely artificial molecules developed to interact with the endogenous cannabinoid system.

Synthetic and semi-synthetic cannabinoids represent a large and heterogeneous group of compounds that currently comprise more than 190 substances reported to the European Monitoring Centre for Drugs and Drug Addiction (7). They are considered the most dangerous class of cannabinoid as they act as a potent full agonist at cannabinoid receptors, producing prolonged effects and more severe adverse reactions compared to those of THC (8). Although precise data on the global prevalence of synthetic and semi-synthetic cannabinoids remain scarce, their presence in illicit markets is expanding, and they now represent the largest group of compounds among the new psychoactive substances (NPS) (9), a broad range of synthetic or plant-derived substances designed to mimic the pharmacological effects of established illicit drugs while circumventing legal restrictions and drug scheduling frameworks.

The psychoactive properties of exogenous cannabinoids are primarily mediated by activation of endogenous cannabinoid receptor CB1, which is widely expressed in the central nervous system (CNS) and modulates the release of neurotransmitters (such as, GABA, glutamate, dopamine) (10). Exogenous cannabinoids also act on CB2 receptors (11–13), which are predominantly expressed in the immune system but are also present at lower levels on neurons and glial cells, including astrocytes, which provide essential structural and metabolic support to neurons (14,15), and microglial cells, which represent up to 20% of the non-neuronal CNS cell population and function as the resident immune effectors of the brain (16).

The distribution of cannabinoid receptors on glia cells, particularly CB2, suggest that the endocannabinoid system plays a crucial role in neuroinflammation, which is defined as an inflammatory response within the CNS (17). Although oligodendrocytes and other glial populations may express components of the endocannabinoid system, this review focuses on microglia and astrocytes as the principal mediators of neuroinflammatory and homeostatic processes in the CNS, given their predominant involvement in cannabinoid-induced modulation of inflammatory signaling pathways. In particular, neuroinflammatory processes are largely mediated by microglia, which can adopt either anti-inflammatory or pro-inflammatory phenotypes in response to insults such as ischemia, trauma, or infections by releasing cytokines and neurotoxic mediators (18,19). Dysregulated or chronic microglial activation leads to persistent neuroinflammation, contributing to neurogenerative process, as observed in multiple sclerosis (MS), Alzheimer disease (ad), and HIV-associated dementia (20–23). In 2023 approximately 2.9 million people worldwide were affected by MS (24). IN 2021 approximately 57 million individuals globally suffered from dementia; of these, 60–70% are estimated to have ad (25). HIV-associated dementia also remains a significant concern in people living with HIV, with prevalence estimates ranging from 16 to 22% (26,27).

Considering both the widespread use of cannabinoids in the general population and the high incidence of neurodegenerative diseases, it is particularly relevant to clarify the long-term effect of cannabinoids and determine whether and how they can be used to modulate glial activation for therapeutic application in the context of neuroinflammatory and neurodegenerative disorders.

## Methods

Following JBI guidelines, this analysis was conducted by a multidisciplinary team of forensic pathologists, a toxicologist and a psychiatrist and it was guided by the following research question: “What are the cannabinoid-induced alterations in microglia and astrocytes and how do these changes modulate neuroinflammatory processes?”

### Information sources and searching

To identify suitable keywords, one researcher (blinded for peer review) conducted an initial search in PubMed. Based on the results, the following search string was developed and refined: (microglia OR “microglial cells” OR neuroglia OR “glial cells”) AND (cannabis OR cannabinoids OR tetrahydrocannabinol OR THC OR CBD OR marijuana) AND (neuroinflammation OR “neuroinflammatory diseases” OR “central nervous system diseases” OR neurodegeneration OR neurotoxicity OR neuroimmune) AND (alterations OR changes OR effects OR dysfunction OR activation OR impairment). The search string was then modified for three databases: PubMed, Scopus and Web of Science. The protocol for this review was registered on the Open Science Framework ([OSF], blinded for peer review).

### Study selection

**Figure 1:**
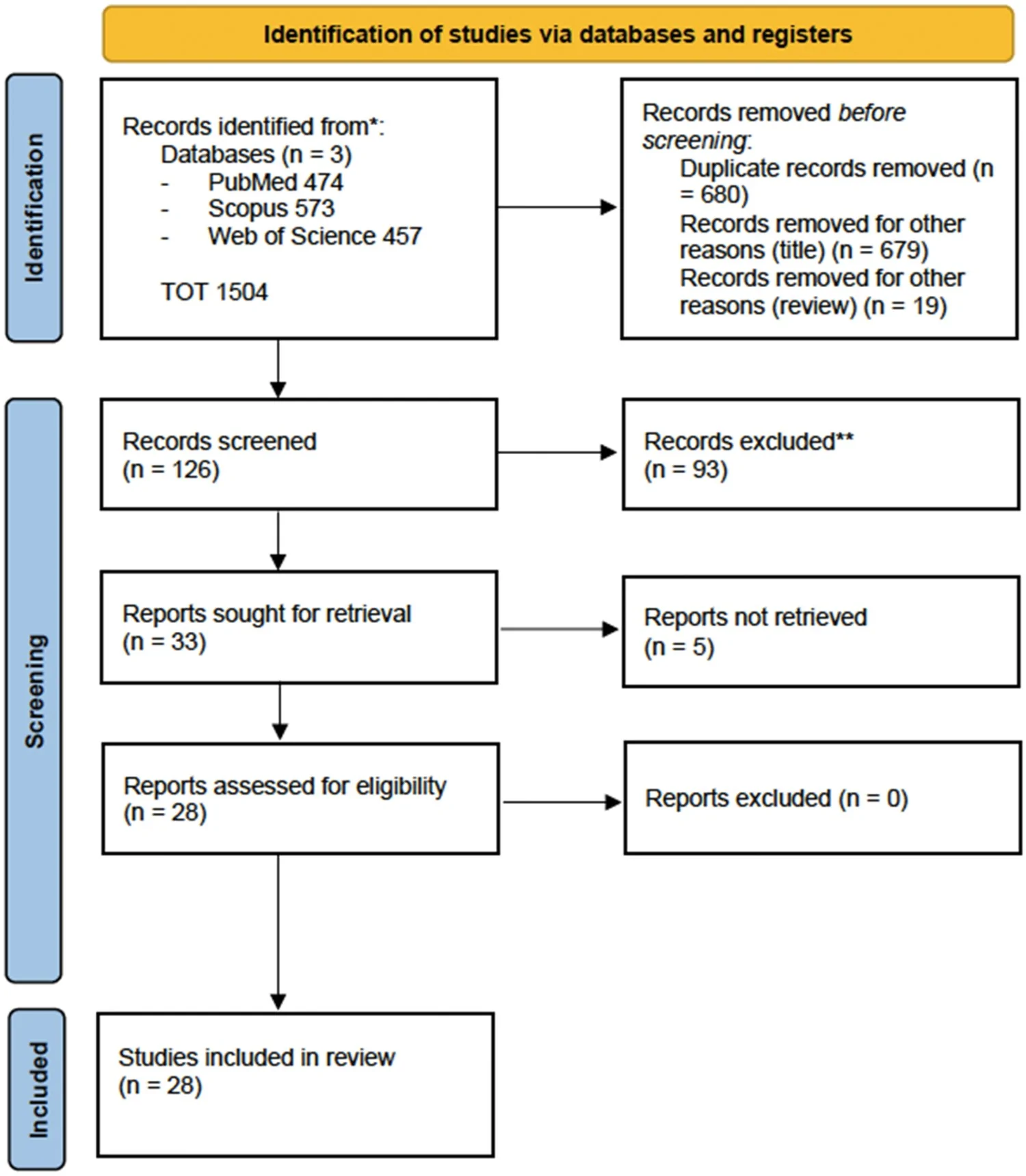
Prisma flow-chart: search results.

All identified references were downloaded in CSV format and uploaded to the online systematic review platform Rayyan, where duplicate articles were automatically identified and manually removed. Two independent researchers (blinded for peer review) screened titles and abstracts according to predefined inclusion and exclusion criteria (Table 1). The search results and the studies included and excluded are fully reported in Figure 1, following PRISMA-ScR extension guidelines. No timeframe was applied.

**Table 1:** Inclusion and exclusion criteria.

*Inclusion Criteria*
**1** Studies that clearly focus on exogenous cannabinoids’ effects on microglial cells and astrocytes.
**2** Language: publications in English

*Exclusion Criteria*
**1** Studies on the effects of exogenous cannabinoids when used alongside other drugs.
**2** Secondary data, conference abstracts, letters, editorials, opinion papers and gray literature.

### Data extraction and analysis

The full-text assessment was conducted by two independent researchers (GBr and GBe) who developed an extraction table that includes the paper that fulfilled all the inclusion criteria for this study (Table 2). The table is composed of the title, first author’s surname, journal, year of publication, study type (vitro/vivo) and model, analysis type (molecular/histological/immunohistochemical), type of exposure (acute/sub-chronic/chronic) and main results.

**Table 2:** Main characteristics of the included studies (*n = *28).

First author Year of publication Title Reference number	Journal	Study type and model	Analysis type	Type of exposure	Main results
di Giacomo V. 2020 Antioxidant and neuroprotective effects induced by cannabidiol and cannabigerol in rat CTX-TNA2 astrocytes and isolated cortexes. (28)	*International Journal of Molecular Science*	In vitro: murine astrocytes CTX-TNA2 Ex vivo: rat brain cortex	• Molecular	Acute (3 hours)	• In vitro: CBD and CBG mitigate astrocytes apoptosis related to oxidative stress (induced by H2O2 treatment). The study suggests this could be by both decreasing the production of reactive oxygen species (ROS) through inhibition of NADPH oxidase and increasing the antioxidant response via activation of the Nrf2/HO-1 pathway. • Ex vivo: CBD (CBG minor) restores levels of metabolites of tryptophan (3‑OH‑Kynurenine and Kynurenic) which play roles in neuroprotection. The proteomic analysis and molecular docking underline how these results depend on the interaction between CBD/CBG and NK3R (neurokinin B receptor) and not with CB1 or CB2 receptors.
Cutando L. 2013 Microglial activation underlies cerebellar deficits produced by repeated cannabis exposure. (29)	*The Journal of Clinical Investigation*	In vivo: murine model (male adults)	• Molecular • Histological • Immunohistochemical	Sub chronic (6 days)	THC chronic exposure causes downregulation of CB1 receptors in cerebellum, leading to increased neuronal excitability and consequently microglial activation (as evidenced by phenotypical changes and elevated expression of CD11b, an adhesion molecule). Activated microglial cells increase expression of CB2 receptor and release pro-inflammatory cytokine IL-1β, resulting in neuroinflammation which clinically manifests as deficits in motor coordination and learning.
Juknat A. 2016 Anti-inflammatory effects of the cannabidiol derivative dimethylheptyl-cannabidiol—studies in BV-2 microglia and encephalitogenic T cells. (30)	*Journal of Basic and Clinical Physiology and Pharmacology*	In vitro: murine microglial cells BV-2 and encephalitogenic T cells	• Molecular	Acute (2 hours)	In microglial cells acute administration of DMH-CBD reduces neuroinflammation both under basal conditions and following LPS-induced inflammation. This effect is mediated by both a decrease in the expression and production of pro-inflammatory cytokines (such as IL-1β, IL-6, and TNF-α) and by the upregulation of antioxidant genes (such as Hmox-1, Trb3, Slc7a11, Atf4, Chop, p8).
Facchinetti F. 2003 Cannabinoids ablate release of TNFalpha in rat microglial cells stimulated with lipopolysaccharide. (31)	*Glia*	In vitro: primary murine microglial cells isolated from the cortex of neonatal rats	• Molecular	Acute (2 hours)	Both endogenous and synthetic (WIN 55,212-2, CP 55,940, HU210) cannabinoids inhibit the release of the pro-inflammatory cytokine TNF-α in microglial cells stimulated with LPS. This suggests cannabinoids reduce neuroinflammation by directly modulating microglia activity, independently by linkage with CB1 and CB2 receptors. The authors propose the existence of yet unidentified cannabinoid receptors mediating this effect.
Coccini T. 2021 MAM-2201, One of the Most Potent-Naphthoyl Indole Derivative-Synthetic Cannabinoids, Exerts Toxic Effects on Human Cell-Based Models of Neurons and Astrocytes. (32)	*Neurotoxicity Research*	In vitro: human cell-based models of neurons and astrocytes derived from humans induced pluripotent stem cells (hiPSCs)	• Molecular • Immunohistochemical	Acute (3 to 24 to 48 hours)	MAM-2201 increases astrocytic production of pro-inflammatory cytokines (such as IL-6) and ROS and elevates Caspase-3/7 activity, leading to neuronal damage and neuroinflammation. Indeed, neuronal cells exposed to MAM-2201 show reduced expression of MAP-2 and NSE expression (fundamental proteins for neuronal morphology and function) resulting in morphological alterations.
Rizzo M.D. 2019 Δ9-Tetrahydrocannabinol Suppresses Monocyte-Mediated Astrocyte Production of Monocyte Chemoattractant Protein 1 and Interleukin-6 in a Toll-Like Receptor 7–Stimulated Human Coculture. (33)	*The Journal of Pharmacology and Experimental Therapeutics*	In vitro: human coculture model consisting of human monocytes derived from peripheral blood and primary human astrocytes	• Molecular	Acute (24 hours)	Δ9-THC, through activation of the CB2 receptor, reduces both the transcription of the pro-inflammatory cytokine gene IL-1β and its maturation by decreasing caspase-1 activity (which converts pro-IL-1β into active IL-1β) in monocytes stimulated with TLR7. As a result, astrocytes—which are normally activated by monocyte-derived signals—reduce their production of other pro-inflammatory cytokines such as IL-6 and MCP-1, leading to a decrease in neuroinflammation.
De Simone U. 2023 Human Astrocyte Spheroids as Suitable In Vitro Screening Model to Evaluate Synthetic Cannabinoid MAM2201-Induced Effects on CNS. (34)	*International Journal of Molecular Science*	In vitro: human astrocyte spheroids	• Molecular • Histological • Immunohistochemical	Acute (24 to 48 hours)	Synthetic cannabinoid MAM2201 by acting on CB1 receptor exerts cytotoxic effect on astrocytes as evidenced by decrease in cell proliferation (which reflect increased caspase-3/7 activity) and morphological changes (associated with reduction in E-cadherin and GFAP—Glial Fibrillary Acidic Protein). By damaging astrocytes, MAM2201 contributes to the development of neuroinflammation.
Landucci E. 2022 Neuronal and Astrocytic Morphological Alterations Driven by Prolonged Exposure with Δ9-Tetrahydrocannabinol but Not Cannabidiol. (35)	*Toxics*	In vitro: organotypic murine hippocampal slices	• Molecular • Histological • Immunohistochemical	Chronic (2 weeks)	Prolonged THC, but not to CBD, incubation causes modifications in the expression of synaptic proteins and morphological alterations in neurons and astrocytes (such as clasmatodendrosis and reduced GFAP expression), which are usually associated with pathological condition including neuroinflammation. The study does not directly measure markers of neuroinflammation; inflammation is only hypothesized as one of the potential mechanisms underlying the observed changes.
Ehrhart J. 2005 Stimulation of cannabinoid receptor 2 (CB2) suppresses microglial activation. (36)	*Journal of Neuroinflammation*	In vitro: primary murine microglial cells	• Molecular	Acute (< 24 hours)	Activation of the microglial CB2 receptor by JWH-015 exerts anti-inflammatory effect: inhibits JAK/STAT1 pathway and consequently the expression of gene such as CD40 (a TNF receptor superfamily member which promotes cytokine production when activated), iNOS (an enzyme responsible for Nitric oxide synthesis) and TNF-α (pro-inflammatory cytokine).
Wang S. 2019 Cannabisin F from Hemp (Cannabis sativa) Seed Suppresses Lipopolysaccharide-Induced Inflammatory Responses in BV2 Microglia as SIRT1 Modulator. (37)	*International Journal of Molecular Science*	In vitro: murine microglial cells BV-2	• Molecular	Acute (< 24 hours)	Cannabisin F exerts anti-neuroinflammatory and antioxidant effects on microglial cells. It enhances SIRT-1 expression blocking NF-kB pathways and, consequently, reducing mRNA levels of pro-inflammatory cytokines (such as IL-6 and TNF-α), and promotes the activation of Nrf2 (Nuclear factor erythroid-2 related factor 2) and HO-1 (Heme Oxygenase-1) reducing the production of ROS.
Pintori N. 2024 Immune and glial cell alterations in the rat brain after repeated exposure to the synthetic cannabinoid JWH-018. (38)	*Journal of Neuroimmunology*	In vivo: murine model	• Molecular • Immunohistochemical	Chronic (2 weeks)	Repeated JWH-018 exposure (administration once daily for 14 days with analyses conducted during the withdrawal period, 7 days after the end of treatment) induces glial cell activation in striatum, evidenced by increased cellular density of both GFAP-positive astrocytes and IBA-1 positive microglia. Consequently JWH-018 increases the production of inflammatory cytokines (IL-2, IL-4, IL‑12p70, IFN‑γ) suggesting its role in neuroinflammation. The study doesn’t analyze which receptors mediates these effects.
Yang M. 2024 The phenylpropionamide extract of hemp (Cannabis sativa L.) seed improves learning memory ability and survival rate by attenuating microglia activation and reducing amyloid deposition via the SIRT1-AMAD10 pathway in APP/PS1 mice. (39)	*Phytomedicine Plus*	In vivo: murine model of Alzheimer (transgenic rats APPswe/PS1dE9)	• Molecular • Histological • Immunohistochemical	Chronic (13 weeks)	After TPA administration (once daily for 13 weeks), hippocampal neurons in the CA1 and CA3 regions exhibit improved morphology and reduced apoptosis, suggesting that TPA exerts neuroprotective effects. This protection appears to be mediated by a reduction in microglial activation, as evidenced by a decrease in IBA-1-positive cells. The diminished microglial reactivity is associated with upregulated SIRT1 expression, leading to a reduction in pro-inflammatory cytokines (such as TNF-α, IL‑1β, and IL‑6) and an increase in antioxidant enzyme activity.
Rodrigues FDS 2024 Cannabidiol prevents LPS-induced inflammation by inhibiting the NLRP3 inflammasome and iNOS activity in BV2 microglia cells via CB2 receptors and PPARγ . (40)	*Neurochemistry International*	In vitro: murine microglial cells BV-2	• Molecular • Immunohistochemical	Acute (2 hours)	CBD exerts an anti-inflammatory effect on microglial cells activated by LPS: by suppressing the iNOS and NLRP3/Caspase-1 pathway it reduces NO and IL‑1β, respectively. The inhibition of NO is mediated exclusively through activation of the PPARγ receptor, while the reduction of IL‑1β involves both PPARγ and CB2 receptors. Additionally, CBD decreases TNF‑α production via a mechanism independent of PPARγ and CB2 receptor activation.
Dos Santos Pereira M. 2023 The two synthetic cannabinoid compounds 4′-F-CBD and HU-910 efficiently restrain inflammatory responses of brain microglia and astrocytes. (41)	*Glia*	In vitro: murine microglial cell and astrocytes	• Molecular • Immunohistochemical	Acute (24 hours)	The synthetic cannabinoids 4′-F-CBD and HU-910 attenuate LPS-induced glial activation, as evidenced by the reduced cellular density of GFAP-positive astrocytes and Iba1-positive microglia. This is accompanied by a significant decrease in pro-inflammatory cytokines (IL‑1β, IL‑6, and TNF‑α), indicating a potential neuroprotective effect. The study highlights that these effects are independent of CB1, CB2, GPR55, and PPARγ receptors, and are instead attributed to the compounds’ antioxidant activity, specifically through inhibition of NOX2 assembly, leading to reduced ROS production and downstream NF-κB signaling
Young A.P. 2022 Synthetic cannabinoids reduce the inflammatory activity of microglia and subsequently improve neuronal survival in vitro. (42)	*Brain, Behavior, and Immunity*	In vitro: murine microglial cell	• Molecular	Acute (16 to 72 hours)	The synthetic cannabinoids ACEA (selective for CB1 receptors) and HU-308 (selective for CB2 receptors) attenuate the pro-inflammatory response of microglia (induced by LPS INF-γ) suppressing MAPK signaling pathway and consequently reducing the release of nitrite oxide (NO) and pro-inflammatory cytokines (IL‑1β, IL‑6, and TNF‑α). The synthetic cannabinoid CP 55.940 (non-selective) have similar effects but was less potent compared to the selective CB1 and CB2 agonists.
Borgonetti V. 2022 Non‐psychotropic Cannabis sativa L. phytocomplex modulates microglial inflammatory response through CB2 receptors‐, endocannabinoids‐, and NF‐κB‐mediated signaling. (43)	*Phytotherapy Research*	In vitro: murine microglial cells BV-2	• Molecular	Acute (24 hours)	The LPS-induced upregulation of the pro-inflammatory cytokines IL‑1β, IL‑6, and TNF‑α was attenuated by CSE, a Cannabis sativa extract enriched in CBD and terpenes, suggesting a neuroprotective effect. These anti-inflammatory effects were only partially dependent on CB2 receptor and were also mediated by the inhibition of reactive oxygen species (ROS) release and the modulation of JNK/p38 cascade with consequent NF-kB p65 nuclear translocation suppression.
Henriquez J.E. 2020 Δ9-Tetrahydrocannabinol (THC) Impairs CD8+ T Cell-Mediated Activation of Astrocytes. (44)	*Journal of Neuroimmune Pharmacology*	In vitro: human coculture of CD8+ T-cells and U251 astrocytes	• Molecular	Acute (72 to 96 hours)	Normally, CD8+ T cells contribute to neuroinflammation by secreting cytokines (such as IFNγ and TNF‑α), which stimulate astrocytes to release additional inflammatory cytokines (such as MCP-1, IP-10, IL-6). THC counteracts neuroinflammation by reducing certain CD8+ T cell responses—particularly those leading to IFNγ production—that contribute to astrocyte activation. Treatment with THC reduced CD8+ T cell-mediated induction of IP-10 and IL-6 responses. The study doesn’t identify the receptor and the signaling pathway through which TCH acts on CD8+ T cells.
Wu J. 2021 Studies of involvement of G-protein coupled receptor-3 in cannabidiol effects on inflammatory responses of mouse primary astrocytes and microglia. (45)	*PLOS One*	In vitro: murine microglial cells and astrocytes In vivo: murine model	• Molecular • Immunohistochemical	Acute (20 hours)	CBD reduces IL-6 release in both LPS-activated astrocytes and microglia in vitro, and TNF‑α release in microglia. In astrocytes, this effect is associated with the inhibition of NF-kb p65 and STAT3 phosphorylation (inducted by LPS activation). In vivo, CBD reduces the mRNA expression of IL-1β and TNF‑α in both astrocytes and microglia cells isolated from the brains of LPS-treated mice. These results suggest a neuroprotective, anti-inflammatory, effect of CBD. Previous studies demonstrate CBD low affinity for CB1 and CB2 receptors. Therefore, this article investigates the role of G protein coupled receptor 3 (GPR3), a putative target. However, the anti-inflammatory effects of CBD on cytokine release are shown to be independent of GPR2, as they persist in GPR3-deficient cells.
Cardinal von Widdern J. 2020 Abnormal Cannabidiol Affects Production of Pro-Inflammatory Mediators and Astrocyte Wound Closure in Primary Astrocytic-Microglial Cocultures. (46)	*Molecules*	In vitro: murine coculture of astrocytes and microglial cells	• Molecular • Histological • Immunohistochemical	Acute (18 to 24 hours)	Abn-CBD, a synthetic isomer of CBD with low affinity for CB1 and CB2 receptors, reduced the production of pro-inflammatory mediators, such as TNF‑α and NO, but doesn’t affect IL-6 levels in astrocyte-microglia cocultures. This suggests an anti-inflammatory effect of Abn-CBD, which is retained even in CB2 knockout cultures, indicating that its mechanism of action is independent of CB2 receptor signaling.
Dos Santos Pereira M. 2020 Cannabidiol prevents LPS-induced microglial inflammation by inhibiting ROS/NF-κB-dependent signaling and glucose consumption. (47)	*Glia*	In vitro: murine microglial cells	• Molecular	Acute (24 hours)	CBD inhibits the release of proinflammatory cytokines (TNF-α and IL-1β) and of glutamate, a noncytokine mediator of inflammation. The main mechanism underlying its neuroprotective effect involves the suppression of ROS production through the inhibition of NADPH oxidase. This action appears to be independent of cannabinoid receptor signaling.
Chung E.S 2012 Cannabinoids prevent lipopolysaccharide-induced neurodegeneration in the rat substantia nigra in vivo through inhibition of microglial activation and NADPH oxidase. (48)	*Brain Research*	In vivo: murine model.	• Molecular • Histological • Immunohistochemical	Subchronic (7 days)	In microglial cells activated by LPS, the synthetic cannabinoids WIN55, 212-2 and HU210 reduce the activation of NADPH oxidase, a key enzyme responsible for ROS production. In addition, they decrease the release of proinflammatory cytokines (TNF‑α and IL-1β). Both ROS and proinflammatory cytokines contribute to dopaminergic neuronal death; indeed histological (Nissl staining) and histochemical (quantification of tyrosine hydroxylase-positive neurons) analysis shows that these cannabinoids attenuate LPS-induced neuronal death in the substantia nigra. Together, these results support the anti-inflammatory and neuroprotective effect of WIN55, 212-2 and HU210. The receptor-mediated mechanism is not explicitly defined.
More S.V. 2013 Anti-neuroinflammatory activity of a novel cannabinoid derivative by inhibiting the NF-κB signaling pathway in lipopolysaccharide-induced BV-2 microglial cells. (49)	*Journal of Pharmacological Sciences*	In vitro: murine microglial cells BV-2	• Molecular • Histological • Immunohistochemical	Acute (24 hours)	The cannabinoid derivate CD-101 exert an anti-inflammatory effect in LPS-activated microglial cells by inhibiting intracellular signaling pathways (such as p38 MAPK phosphorylation and NF-kb p65 nuclear translocation) and consequently reducing pro-inflammatory mediators (such as NO, COX-2, IL-6, TNF‑α and IL-1β) production.
Froger N. 2009 Cannabinoids prevent the opposite regulation of astroglial connexin43 hemichannels and gap junction channels induced by pro-inflammatory treatments. (50)	*Journal of Neurochemistry*	In vitro: murine cerebral cortex, including astrocytes and microglial cells	• Molecular • Histological • Immunohistochemical	Acute (24 hours)	Cannabinoids (WIN-55, 212-2, CP-55,940 and methanandamide) exert a neuroprotective effect in inflammatory conditions induced by LPS stimulation by: suppressing the release of pro-inflammatory cytokines (such as TNF‑α and IL-1β) from activated microglia and preserving the normal function of CX43 channels in astrocytes which prevent the pathological opening and the closure of gap junctions. These effects are mainly mediated through CB1 receptors, but additional contributions from non-CB11-CB2 pathways, especially in the case of methanandamide, are also suggested.
Martin-Moreno A.M. 2012 Prolonged oral cannabinoid administration prevents neuroinflammation, lowers β-amyloid levels and improves cognitive performance in Tg APP 2576 mice. (51)	*Journal of Neuroinflammation*	In vivo: murine model of Alzheimer transgenic rats (Tg APP 2576)	• Molecular • Immunohistochemical • Imaging	Chronic (5 weeks)	In Tg APP mice there is a significant increase in microglial and astrocytes activation, as indicated by elevated levels of Iba1 and GFAP. Chronic administration of cannabinoid reduces microglial activation but does not affect astrogliosis. This effect appears to be mediated by CB2 receptors because only CB2-selective agonists, such as JWH-133 - and not mix CB1/CB2 agonists such as WIN55, 212-2- produce this outcome. However, both JWH-133 and WIN55,212-2 have anti-inflammatory action demonstrated by decreased levels of pro-inflammatory markers (COX-2 and TNF‑α). The neuroprotective action of JWH-133 is given also by the normalization of glucose uptake, evaluated by ¹⁸F-FDG PET. In addition, both cannabinoids improve the amyloid clearance, as evidenced by Aβ level reduction).
Kozela E. 2011 Cannabidiol inhibits pathogenic T cells, decreases spinal microglial activation and ameliorates multiple sclerosis-like disease in C57BL/6 mice. (52)	*British Journal of Pharmacology*	In vitro: encephalitogenic T cell from immunized mice In vivo: murine model of Multiple Sclerosis (Experimental Autoimmune Encephalomyelitis)	• Molecular • Histological • Immunohistochemical	Acute (72 hours)	Acute administration of CBD at the onset of EAE symptoms slows down disease progression by suppressing microglial and macrophage activation, as evidenced by decrease of Iba-1 and Mac-2 levels, in addition to reducing pathogenic T-cell recruitment in the spinal cord. This suggests neuroimmunomodulatory actions of CBD in multiple sclerosis-like disease models, through mechanisms independent of canonical CB1 and CB2 receptors.
Janefjord E. 2013 Cannabinoid Effects on β Amyloid Fibril and Aggregate Formation, Neuronal and Microglial-Activated Neurotoxicity In Vitro. (53)	*Cellular and Molecular Neurobiology*	In vitro: murine microglial cells BV-2 and neuronal cells SH-SY5Y	• Molecular	Acute (24 hours)	Non-selective CB1/CB2 cannabinoids (such as 2-AG and CBD) significantly reduce TNF‑α and NO production in microglial cells activated with LPS, showing anti-inflammatory effects. Contrarily, selective cannabinoids (such as ACEA—CB1 agonist—and JWH-015 - CB2 agonist) do not significantly reduce TNF‑α and NO production, suggesting that CB1/CB2 receptor activation is not responsible for the anti-inflammatory effects. Additionally, 2-AG and CBD have increased neuronal vitality after administration of Aβ1 to 42, typically associated with cellular death and oxidative stress.
Granja A.G. 2012 A cannabigerol quinone alleviates neuroinflammation in a chronic model of multiple sclerosis. (54)	*Journal of Neuroimmune Pharmacology*	In vitro: murine microglial cells BV-2 In vivo: murine model of Multiple Sclerosis (Experimental Autoimmune Encephalomyelitis)	• Molecular • Histological • Immunohistochemical	Chronic (10 days)	Chronic treatment with VCE-003, a cannabigerol quinone, reduce microglial activation (as evidenced by lower Iba1 levels), production of pro-inflammatory markers (such as TNF‑α, IL-1β, e IFN-γ) and ROS. These anti-inflammatory effects seem to be mediated by PPARγ rather than traditional cannabinoid receptors.
Marchalant Y. 2009 Cannabinoids attenuate the effects of aging upon neuroinflammation and neurogenesis. (55)	*Neurobiology of Disease*	In vivo: murine model	• Molecular • Immunohistochemical	Chronic (4 weeks)	Normally, in aged rats there is a significant increase in microglial activation in the hippocampus, a hallmark of age-related neuroinflammation, along with a decrease in neurogenesis. Chronic treatment with the cannabinoid WIN-2 reduces the density of OX-6- positive microglial cells (a marker of activated microglia), indicating an anti-inflammatory effect, as supported by lower levels of pro-inflammatory cytokines (such as TNF‑α and IL-1β). This effect appears to be mediated by WINS-2’s antagonism of the TRPV1 receptor. Additionally, chronic treatment with WIN-2 increases the density of BrdU- and DCX-positive neuronal cells (markers of proliferation and migration respectively), indicating enhanced neurogenesis This effect seems to depend on WIN-2’s agonist activity at CB1/CB2 receptors. Therefore, the study suggests TRPV1 receptor has a role in anti-inflammation, whereas CB1/CB2 receptor in neurogenesis.

Abbreviation list: 2-AG: 2-Arachidonoylglycerol**;** Atf4: Activating Transcription Factor 4; Abn-CBD: Abnormal Cannabidiol**;** ACEA: Arachidonyl-2’-chloroethylamide**;** CBD: Cannabidiol; CBG: Cannabigerol**;** CBN: Cannabinol**;** CBRs: Cannabinoid Receptors; CD11b: Cluster of Differentiation 11 b**;** CD8: Cluster of Differentiation 8; CSE: Cannabis Sativa Extract; DMH-CBD: Dimethylheptyl Cannabidiol**;** EAE: Experimental Autoimmune Encephalomyelitis; GFAP: Glial Fibrillary Acidic Protein; GPR: G Protein-coupled Receptor; HO-1: Heme Oxygenase-1**;** Iba-1: Ionized Calcium-Binding Adapter Molecule 1; IL-1β: Interleukin-1 β**;** IL-2: Interleukin-2**;** IL-4: Interleukin-4**;** IL-6: Interleukin-6; IL-12p70: Interleukin-12p70**;** INF-γ: Interferon gamma; iNOS: Inducible Nitric Oxide Synthase**;** JAK: Janus kinase; LPS: Lipopolysaccharide**;** MAP-2: Microtubule-Associated Protein 2; MAPK: Mitogen-Activated Protein Kinase; MCP-1: Monocyte Chemoattractant Protein**;** NADPH: Nicotinamide Adenine Dinucleotide Phosphate (reduced form); NF-kB: Nuclear Factor Kappa-light-chain-enhancer of activated B cells**;** NLRP3: NOD-like receptor family, pyrin domain containing 3**;** NO: Nitric oxide**;** NOX2: NADPH oxidase 2; Nrf2: Nuclear factor erythroid 2-related factor 2**;** PPARγ: Peroxisome Proliferator-Activated Receptor Gamma**;** ROS: Reactive Oxygen Species**;** SIRT-1: Sirtuin 1; Slc7a11: Solute Carrier Family 7 Member 11**;** STAT: Signal Transducer and Activator of Transcription; THC: Δ9-tetrahydrocannabinol; TLR7: Toll-like Receptor 7; TNF-α: Tumor Necrosis Factor α; TPA: Total phenylprpionamide; Trb3: Tribbles Psuedokinase 3; TRPV1: Transient Receptor Potential Vanilloid 1.

## Results

### Search results

Initially, the search string identified 1504 studies. After duplicate removal and screening of titles and abstracts, 33 articles were selected for full-text assessment. Of these, 28 met the inclusion criteria and were therefore included in the review (Figure 1).

### Study characteristics

In most of the articles, studies were conducted only in vitro (*n = *18), with a predominance of murine models compared to human models (*n = *14 and *n = *4, respectively); the remaining articles include studies conducted only in vivo (*n = *6), both in vivo and in vitro (*n = *3), and in vitro and ex vivo (*n = *1), all using murine models.

**Figure 2:**
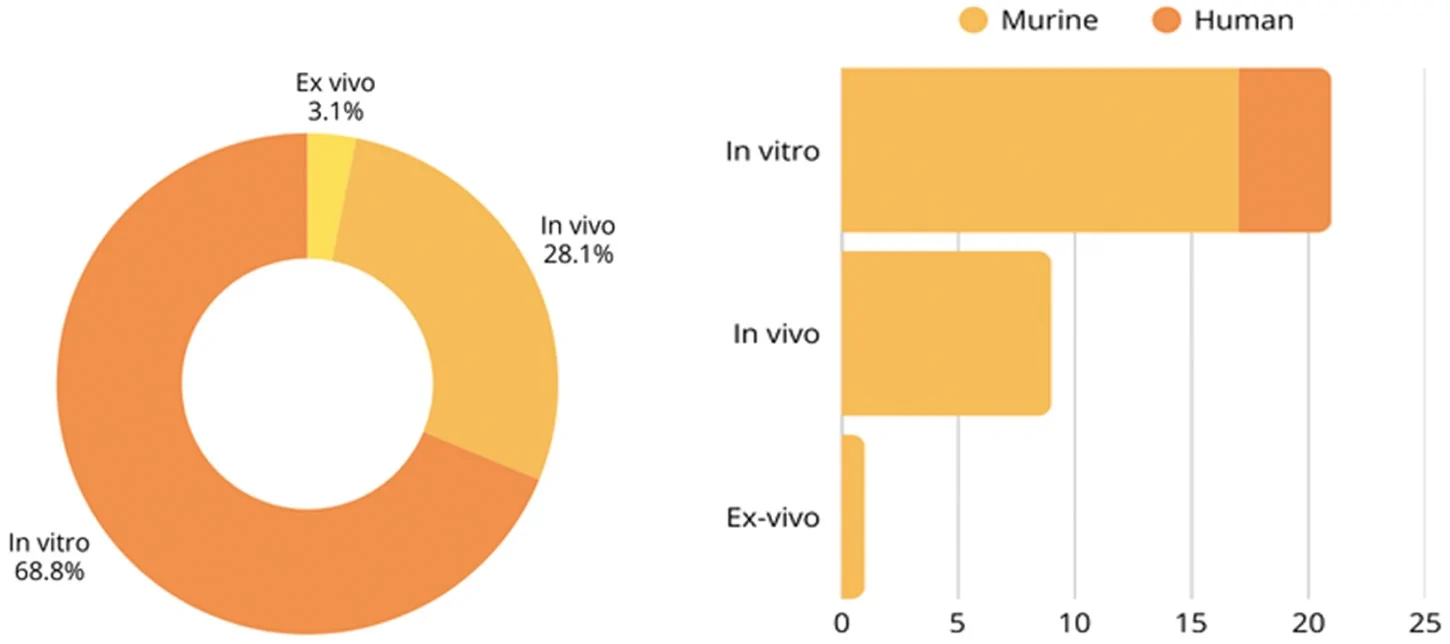
Distribution of study types and model organism among the articles included.

Molecular analysis was performed in all the articles; histological and immunohistochemical analyses were reported only in a subset (*n = *10 and *n = *17, respectively) (Figure 2).

**Figure 3:**
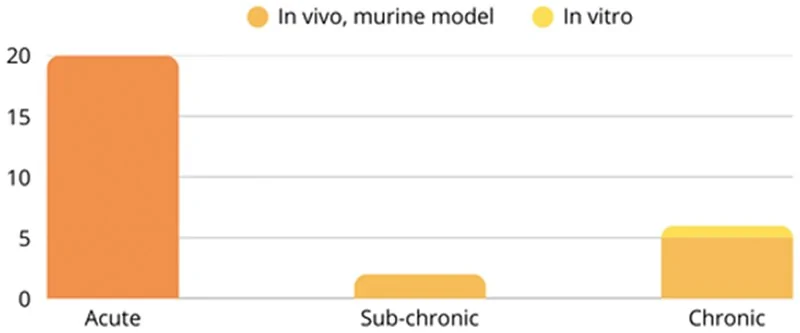
Distribution of type exposure among the articles included.

Most of the studies analyze the effects of acute cannabinoid exposure (*n = *20); chronic and sub-chronic (6–7 days) exposures are evaluated only in a minority (*n = *6 and *n = *2, respectively), with all but one conducted in vivo using a murine model (Figure 3).

“Acute exposure” was defined as short-term cannabinoid treatment ranging from 1 to 72 hours. In in vitro studies, this corresponded to the incubation time of cultured cells with cannabinoids; in in vivo studies it denoted a single administration or, in some cases, repeated administrations over a short time window within the acute period.

“Sub-chronic exposure” and “chronic exposure” were defined as repeated or continuous exposure lasting from several days up to approximately one week and beyond one week, respectively. As regards the term “sub-chronic,” in one article (29), mice were analyzed five days after exposure, while in another article (48) the animals were killed after seven days.

It is important to stress that the exposure categories (acute, sub-chronic and chronic) were defined as functional experimental descriptors based on the duration of treatment within each individual study design, independently of the experimental system (in vitro murine, in vitro human, in vivo murine). Therefore, these categories do not represent cross-species temporal equivalence but rather standardized descriptors of short-intermediate-, and long-term cannabinoid exposure within each model.

## Major findings

### Phytocannabinoids

**Figure 4:**
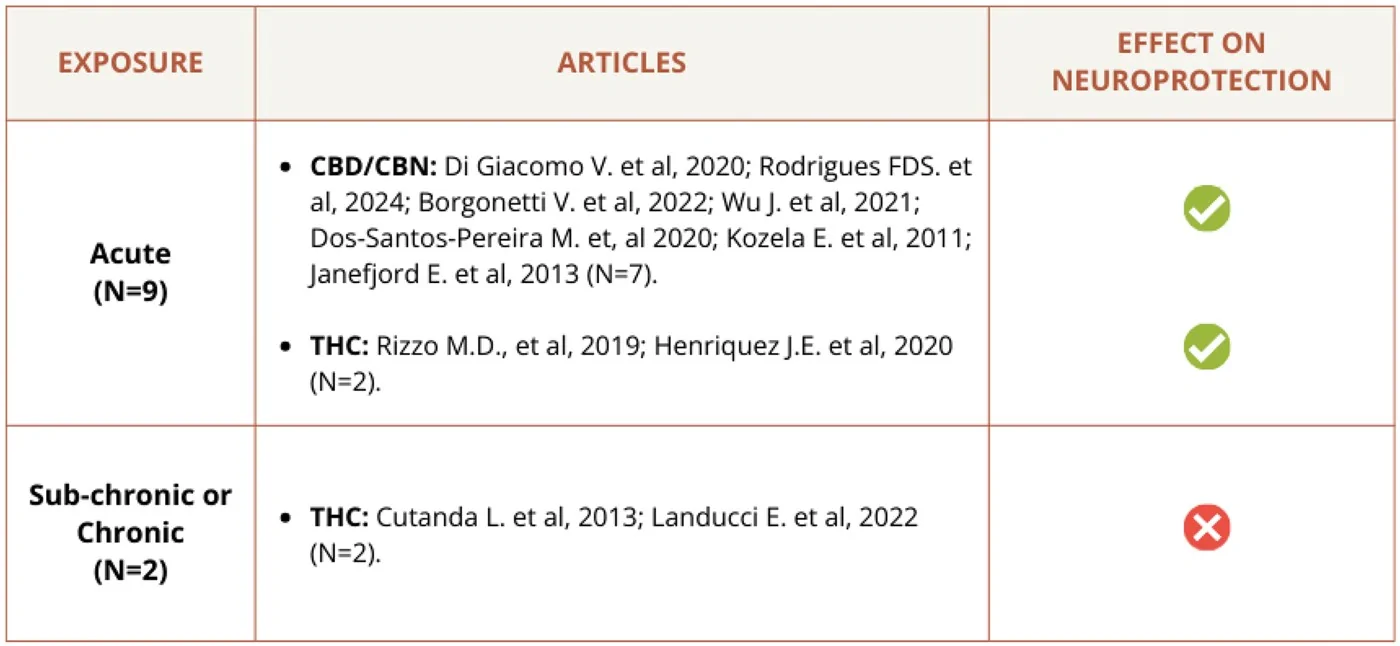
Effects of phytocannabinoids on microglia following acute, sub-chronic and chronic exposure.

Eleven articles investigate the effects of phytocannabinoids on microglial cells and astrocytes following acute (*n = *9), sub-chronic (*n = *1) and chronic (*n = *1) exposure (Figure 4).

#### Acute exposure

Among those on acute exposure, seven studies focused on non-psychotropic phytocannabinoids (CBD and CBN); two focused on psychotropic compound (THC). The totality of articles consistently highlights its neuroprotective effects.

For non-psychotropic compounds, acute exposure appears to directly suppress microglial activation induced by inflammatory stimuli (eg lipopolysaccharide [LPS] stimulation). This is supported by immunohistochemical findings showing reduced expression of Iba-1 and Mac-2 (markers of microglial activation) levels (52). Additionally, five articles observe a decrease in pro-inflammatory cytokines including IL-1β-, IL-6- and TNF-α-release (40,43,45,47,53). One study attributes this effect to the suppression of the iNOS and NLRP3/Caspase-1 pathway (40); another attributes this to the inhibition of NF-kB and STAT3 phosphorylation (45); two studies attribute this to the reduction of ROS production via the inhibition of NADPH oxidase (28,47). Overall, these findings indicate that the neuroprotective effects of acute CBD and other non-psychotropic phytocannabinoids are mediated by direct anti-inflammatory and antioxidant actions on microglia and astrocytes, which appear to be largely independent of canonical CB1 and CB2 receptor activation (28,43,45,47,52,53).

One study shows that CB2 receptor activation by THC suppresses both IL-1β transcription and capsase-1-dependent maturation in monocytes; consequently, astrocytes, which are activated by monocytes release fewer pro-inflammatory mediators (33). Another article suggests that THC reduces CD8+T cell response, particularly IFN-γ production, leading to decreased astrocyte-derived cytokines (44).

### Sub-chronic and chronic exposure

Only THC effects are analyzed after sub-chronic (6 days) and chronic exposure in two distinct articles, which provide evidence that prolonged use may induce pro-inflammatory and neurotoxic effects.

Sub-chronic THC exposure leads to downregulation of CB1 receptors, resulting in increased neuronal excitability and subsequent microglial activation (29). This is reflected by phenotypic changes and upregulation of CD11b (a marker of microglial reactivity), which in turn promotes the release of pro-inflammatory cytokines. In addition to microglial-mediated mechanisms, chronic THC exposure induces structural and synaptic alterations in astrocytes (35), as evidenced with reduced glial fibrillary acidic protein (GFAP) expression (a marker of astrocytes’ integrity and activity) and increased clasmatodendrosis (a degenerative process of astrocytes characterized by swelling, vacuolization and fragmentation of cellular processes, typically associated with severe CNS injury such as ischemia and hypoxia).

### Synthetic cannabinoids

**Figure 5:**
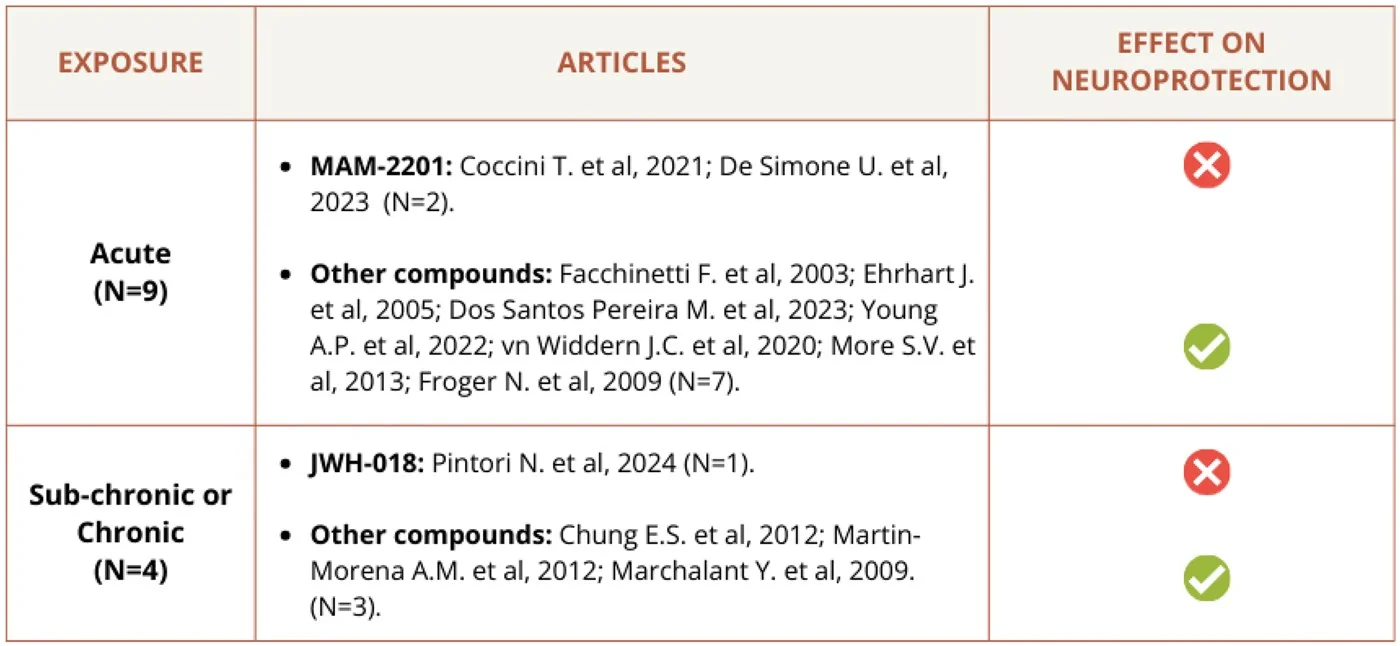
Effects of synthetic cannabinoids on microglia following acute, sub-chronic and chronic exposure.

Thirteen articles investigate the effects of synthetic cannabinoids on microglial cells and astrocytes following acute (*n = *9), sub-chronic (*n = *1) and chronic (*n = *3) exposure (Figure 5).

#### Acute exposure

Most studies on acute exposure to synthetic cannabinoids report a neuroprotective effect, except for two articles on MAM-2201. Acute exposure to MAM-2201 induces CB1 receptor-mediated neurotoxicity, causing astrocyte damage characterized by decreased proliferation, morphological alteration, and increased capsase-3/7 activity. This is accompanied by reduced expression of astrocytes markers (GFAP, E-cadherin) (34) and increased production of pro-inflammatory cytokines (IL-6) and ROS (32). As a result, neuronal cells exhibit decreased levels of key structural and functional proteins (MAP-2 and NSA), with morphological changes which indicate secondary neuronal damage (32).

In contrast, other synthetic compounds (WIN 33,212.2, CP 55,940, HU 210, JWH-015, 4’-F-CBD, HU 910, ACRA, HU-308, CP 55.940, Abn-CBD, CD 101, WIN-55,212-2, CP-55,949), similar to phytocannabinoids, appear to directly suppress microglial activation. This is evidenced by immunohistochemical reductions in GFAP and Iba-1 (41), leading to decreased production and release of pro-inflammatory cytokines–including IL-1β, IL-6, and TNF-α – as reported in all seven articles (31,36,41,42,46,49,50). Most studies suggest that these neuroprotective effects are largely independent of canonical CB1 and CB2 receptor activation (31,41,46). Two studies propose that the inhibition of the MAPK pathway may underlie the effects of ACRA, HU-308, CP 55.940, and CD-101 (42,49). Instead, one study attributes the neuroprotective effect of JWH-015 to CB2 receptor-mediated inhibition of JAK/STAT1 pathway (36).

#### Sub-chronic and chronic exposure

Most articles investigating sub-chronic and chronic exposure to synthetic cannabinoids (WIN 55, 212-2, HU 210, JWH-133, WIN 2) report neuroprotective effects. These compounds appear to suppress microglial activation, as indicated by reduced OX-6 (a marker of activated microglial) immunoreactivity following WIN-2 chronic exposure, and consequently decrease the production of pro-inflammatory cytokines—IL-1β and TNF-α – (48,51,55). The neuroprotective effect of WIN-2 appears to be independent of CB1/CB2 receptor activation and may be mediated by its antagonism at the TRPV1 receptor (55).

In contrast, one article reported that chronic exposure to JWH-018 induces glial cell activation, as evidenced by increased of GFAP and Iba-1 immunoreactivity, with a concomitant rise in pro-inflammatory cytokines (such as, IL-2, IL-4, IL-12p70, IFN-γ) (38).

### Semi-synthetic cannabinoids

**Figure 6:**
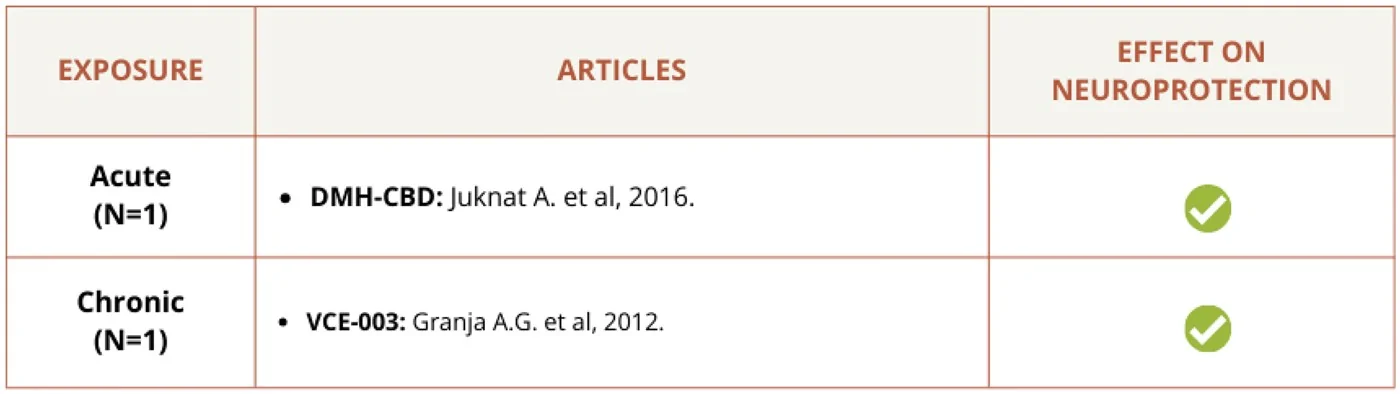
Effects of semi-synthetic cannabinoids on microglia following acute and chronic exposure.

Only two studies have investigated the effects of semi-synthetic cannabinoids on microglial cells and astrocytes. One assessed the impact of acute exposure to dimethyl heptyl-cannabidiol (DMH-CBD) (30), while the other examined the effect of chronic exposure to VCE-003, a cannabigerol quinone derivative (54) (Figure 6).

Both compounds were shown to reduce pro-inflammatory cytokines (such as IL-1β and TNF-α, IL-6) and ROS production, resulting in an anti-inflammatory effect. For VCE-003, these effects appear to be mediated by PPARγ rather than canonical cannabinoid receptors.

## Discussion

This review offers a comprehensive synthesis of the literature on cannabinoid-induced alterations in microglial cells and astrocytes and suggests that these effects are both time-and compound-dependent.

Acute exposure is generally associated with neuroprotective effects: only the synthetic cannabinoid MAM-2201 seems to induce astrocytic and neuronal damage. Instead, acute exposure to others phytocannabinoids, synthetic and semi-synthetic compounds is consistently associated with suppression of glial cell activation, as supported by immunohistochemical findings of reduced expression of GFAP, Iba-1 and Mac-2 levels, as well as with a reduction of pro-inflammatory mediators and ROS. These effects appear to involve diverse molecular pathway (such as iNOS and NLRP3/caspase 1, NF-kB and STAT3, NADPH oxidase, MAPK, JAK/STAT1) independently by CB1 and CB2 receptor activation, carrying important implications as follows: (1) By targeting multiple intracellular pathways, cannabinoids can modulate glial cell reactivity at multiple levels of the inflammatory cascade, which may be advantageous in the treatment of neurodegenerative and neuroinflammatory diseases, where inflammation is not driven by a single mechanism. (2) 45% of the articles (*n = *9 out of a total of 20 studies on acute exposure) reported that beneficial anti-inflammatory and antioxidant effects can occur without the mediation of CB1 receptors, which are known to mediate the psychotropic side effects of cannabinoids or CB2 receptors, which are associated with immune suppression. This suggests the possibility of developing compounds with neuroprotective properties, while minimizing adverse effects on the CNS and immune systems. In this context, Cannabisin F, a phenylpropionamide found in hemp seed, provides a concrete example, as demonstrated in two studies included in this review (37,39). It reduces neuronal apoptosis, enhancing SIRT1 expression, inhibiting NF-kb and promoting Nrf2/HO-1 activation, reducing microglial production of pro-inflammatory cytokines and ROS. These findings underscore the potential of similar compounds to modulate neuroinflammation without engaging the canonical cannabinoid receptors.

It is worth noting that most studies included in this review (68.8%) were conducted in vitro, which limits the translational relevance of current findings. Increasing the number of in vivo studies will be essential to validate the observed mechanism in more complex biological systems.

Chronic exposure remains under-investigated, as it was addressed only in a minority of the studies included in this review (28.6%). Research in this area needs to be expanded to better evaluate the long-term consequences of recreational cannabinoid use and the therapeutic potential in neurodegenerative and neuroinflammatory conditions.

Existing data suggest that most synthetic or semi-synthetic compounds maintain neuroprotective properties during prolonged administration (evidenced by reduced OX-6 immunoreactivity and production of pro-inflammatory cytokines), likely through alternative mechanisms such as TRPV1 antagonism. On the other hand, THC could induce deleterious outcomes in the CNS, such as glial cell activation and/or the release of pro-inflammatory cytokines.

If the deleterious effects of chronic THC exposure on neuroinflammation are confirmed, public health and governing policies should aim to regulate its consumption, particularly among adolescents, not only to reduce the risks associated with the short-term psychoactive effects, but also to prevent long-term consequences, including increased risk of neurodegenerative diseases and neuronal loss. Conversely, given the substantial global burden of neurodegenerative diseases, eg MS, ad and HIV-associated dementia, confirmatory evidence of the neuroprotective effects of cannabinoids would have important public health implications, justifying not only further investment in research but also targeted social and legislative actions. These should include the legalization of therapeutic use in countries where it is not yet permitted and, where it is already allowed, the simplification of rigid prescribing protocols and the implementation of measures to facilitate patient access. It is worth noting that although therapeutic use of cannabinoids is legally permitted in several countries, including Italy, access remains limited due to restricted availability of pharmaceutical-grade preparations, regional disparities in healthcare provision and logistical challenges in production and distribution.

More comprehensive evidence on the acute and chronic effects of cannabinoids including natural, synthetic, and semi-synthetic derivatives could represent a valuable resource for legislative reforms. Such data may contribute both to the refinement of current policies addressing the control of recreational use and to the regulation of patient access to pharmacological therapies based on cannabis preparations.

It is important to note that, although this review distinguishes between “neuroprotective” and “neuroinflammatory” effects according to terminology and interpretations reported in the included studies, recent advances in the field increasingly highlight the limitations of a dichotomous classification, which may not adequately capture the dynamic and context-dependent nature of glial cells. In fact, microglial and astrocytic states are highly plastic and multidimensional. They are equipped with a complex “sensome” of surface receptors that enables continuous sensing of both central and peripheral signals resulting in a dynamic spectrum of states shaped by epigenomic, transcriptomic, proteomic and metabolic programs. In this framework, inflammatory responses in the CNS should not be considered inherently detrimental but rather adaptive processes that may become dysregulated depending on context. Within this perspective, the effects of cannabinoids on glial cells should be interpreted as modulatory actions within a dynamic spectrum of activation states rather than as strictly “neuroprotective” or “neurotoxic” outcomes (56).
